# Mesalazine Nonadherence in Mild-to-Moderate Ulcerative Colitis: Barriers in a Moroccan Cohort

**DOI:** 10.7759/cureus.112781

**Published:** 2026-07-16

**Authors:** Jihane Rizkou, Fatimaezzahra Lairani, Oussama Nacir, Adil Ait errami, Sofia Oubaha, Zouhour Samlani, Khadija Krati

**Affiliations:** 1 Gastroenterology and Hepatology, Mohammed VI University Hospital Center, Marrakech, MAR

**Keywords:** adherence, inflammatory bowel disease, mesalazine, patient education, pill burden, ulcerative colitis

## Abstract

Background: Mesalazine, also known as mesalamine, remains a cornerstone first-line therapy for induction and maintenance of remission in mild-to-moderate ulcerative colitis (UC). However, real-life adherence is frequently suboptimal and may be influenced by behavioral, cultural, therapeutic, financial, and health system-related factors. Data from North African populations remain limited. This study aimed to describe the main determinants of mesalazine nonadherence in a Moroccan cohort of patients with mild-to-moderate UC.

Methods: We conducted a retrospective descriptive study in the Department of Gastroenterology and Hepatology at Mohammed VI University Hospital Center, Marrakech, Morocco, over a two-year period, from September 2023 to September 2025. Consecutive adult patients with mild-to-moderate UC receiving mesalazine were included. Collected variables included demographic characteristics, disease extent, dosing frequency, pill burden, treatment behavior, use of herbal alternatives, fear of medication, forgetfulness, drug availability, financial limitations, and response to targeted counseling. Poor adherence was defined as taking less than 80% of prescribed doses. Data were analyzed using frequencies and percentages.

Results: Thirty patients were included, comprising 23 females and seven males, with ages ranging from 23 to 57 years. Left-sided colitis was the most common disease extent, observed in 18 patients (60%), followed by proctitis in eight patients (26.7%) and pancolitis in four patients (13.3%). Good adherence was reported in 20 patients (66.7%), whereas 10 patients (33.3%) had poor adherence. Among poorly adherent patients, stopping treatment when asymptomatic was the most frequent behavioral factor, reported in six patients (60% of poorly adherent patients; 20% overall). Other factors included use of herbal alternatives in two patients (20%; 6.7% overall), fear of medication in one patient (10%; 3.3% overall), and forgetfulness in one patient (10%; 3.3% overall). High pill burden, defined as at least four tablets per day, was observed in 16 patients (53.3%). Once-daily mesalazine was prescribed in six patients (20%), while 24 patients (80%) received multiple daily dosing regimens. Financial constraints limiting regular drug purchase were reported by eight patients (26.7%), and drug unavailability was reported by one patient (3.3%). Targeted counseling improved adherence in seven of the 10 poorly adherent patients (70%).

Conclusions: Mesalazine nonadherence in this Moroccan UC cohort was frequent and multifactorial. Behavioral factors, cultural beliefs, high pill burden, financial limitations, and health system barriers all contributed to poor adherence. Simplified dosing regimens, improved access to affordable formulations, and structured patient education may help optimize long-term adherence in real-life practice.

## Introduction

Ulcerative colitis (UC) is a chronic inflammatory bowel disease characterized by continuous mucosal inflammation of the colon, typically beginning in the rectum and extending proximally to varying degrees. In patients with mild-to-moderate UC, 5-aminosalicylic acid (5-ASA) therapy, including mesalazine, remains a key first-line therapeutic option for both induction and maintenance of remission [1,2]. Because UC is a chronic relapsing disease, long-term adherence to maintenance therapy is essential to reduce relapse risk and preserve quality of life [3-5].

Despite its established role, mesalazine adherence remains challenging in routine care. Nonadherence may be intentional, such as stopping treatment when symptoms improve, or unintentional, such as forgetting doses or being unable to purchase medication regularly. Previous studies have identified complex dosing schedules, high pill burden, insufficient understanding of maintenance therapy, fear of adverse events, medication cost, and patient beliefs as important contributors to poor adherence [4,6-8]. In settings where drug affordability and availability are variable, these barriers may be amplified.

Evidence on mesalazine adherence is mostly derived from European, North American, and Asian cohorts, while data from North African populations remain limited [6]. In Morocco, adherence may be influenced not only by therapeutic factors such as pill burden and dosing frequency, but also by socioeconomic constraints, access to medication, and culturally rooted practices such as the use of herbal alternatives. Understanding these factors is important for designing practical interventions adapted to local healthcare realities.

This study aimed to describe the frequency and determinants of mesalazine nonadherence in a Moroccan cohort of adults with mild-to-moderate UC, with particular attention to behavioral, cultural, pill-burden-related, financial, and health-system barriers.

## Materials and methods

Study design and setting

We conducted a retrospective descriptive study over a two-year period, from September 2023 to September 2025, in the Department of Gastroenterology and Hepatology at Mohammed VI University Hospital Center, Marrakech, Morocco. Consecutive adult patients followed for mild-to-moderate UC and treated with mesalazine were included.

Study population

Eligible patients were adults aged 18 years or older with a diagnosis of mild-to-moderate UC who were receiving mesalazine as part of their treatment. UC diagnosis and disease extent were based on standard clinical, endoscopic, and histological findings recorded in the medical file. Disease extent was categorized as proctitis, left-sided colitis, or pancolitis.

Exclusion criteria

Patients were excluded if they had severe acute UC requiring hospitalization, Crohn’s disease, indeterminate colitis, incomplete adherence data, or insufficient follow-up information to assess treatment-taking behavior.

Data collection

Data were collected from medical records and follow-up visits. The variables included age range, sex, UC extent, mesalazine dosing frequency, number of tablets per day, use of once-daily versus multiple-dose regimens, drug availability, financial limitations affecting regular purchase, and behavioral or cultural factors associated with non-adherence. Behavioral and cultural factors included stopping treatment when asymptomatic, use of herbal alternatives, fear of medication, and forgetfulness.

Definitions

Poor adherence was defined as taking less than 80% of prescribed mesalazine doses. Good adherence was defined as taking at least 80% of prescribed doses. High pill burden was defined as taking four or more mesalazine tablets per day. Financial limitation was defined as difficulty purchasing mesalazine regularly because of cost. Drug unavailability was defined as patient-reported difficulty obtaining the prescribed formulation.

Adherence assessment

Adherence was assessed retrospectively from medical records and outpatient follow-up notes, based on patient-reported treatment-taking behavior documented by the treating physicians. Poor adherence was defined as taking less than 80% of the prescribed mesalazine doses. Objective adherence measures such as pill counts, pharmacy refill data, electronic monitoring, urinary 5-ASA metabolite testing, or validated adherence questionnaires were not routinely available and were therefore not used.

Targeted counseling

Patients with poor adherence received targeted counseling during follow-up. Counseling focused on explaining the chronic relapsing nature of UC, the role of mesalazine as maintenance therapy even during asymptomatic periods, the risk of relapse after treatment interruption, and the importance of regular dosing. When feasible, dosing simplification and practical advice to reduce missed doses were proposed.

Assessment after targeted counseling

Improvement after targeted counseling was evaluated during subsequent follow-up visits using documented patient-reported treatment-taking behavior and clinician assessment. Improvement was defined as documented regularization of mesalazine intake or return to taking at least 80% of prescribed doses after counseling. No standardized adherence questionnaire was used for postcounseling assessment.

Outcomes

The primary outcome was the frequency of poor adherence to mesalazine. Secondary outcomes included the distribution of behavioral, cultural, pill-burden-related, financial, and health-system factors associated with nonadherence, as well as the proportion of poorly adherent patients who improved after targeted counseling.

Statistical analysis

Descriptive statistics were used. Categorical variables were expressed as frequencies and percentages. Because of the small sample size and descriptive design, no inferential statistical testing was performed.

## Results

Patient characteristics

Thirty adult patients with mild-to-moderate UC receiving mesalazine were included. The cohort comprised 23 women (76.7%) and seven men (23.3%), with ages ranging from 23 to 57 years. Left-sided colitis was the most frequent disease extent, observed in 18 patients (60%), followed by proctitis in eight patients (26.7%) and pancolitis in four patients (13.3%). Baseline characteristics of the study population are summarized in Table 1.

**Table 1:** Baseline characteristics of the study population Values are presented as the number of patients and the percentage unless otherwise specified

Characteristic	Number of patients	Percentage
Total population	30	100%
Female sex	23	76.7%
Male sex	7	23.3%
Age range	23-57 years	-
Proctitis	8	26.7%
Left-sided colitis	18	60%
Pancolitis	4	13.3%

Adherence status

Good adherence was reported in 20 patients (66.7%), whereas poor adherence was observed in 10 patients (33.3%). Thus, approximately one-third of the cohort did not take at least 80% of prescribed mesalazine doses. Mesalazine adherence status is shown in Table 2.

**Table 2:** Mesalazine adherence status Good adherence was defined as taking at least 80% of prescribed mesalazine doses. Poor adherence was defined as taking less than 80% of prescribed doses

Adherence status	Number of patients	Percentage
Good adherence	20	66.7%
Poor adherence	10	33.3%

Behavioral and cultural factors associated with poor adherence

Among the 10 poorly adherent patients, stopping mesalazine when asymptomatic was the most frequent behavioral factor, reported in six patients (60% of poorly adherent patients; 20% of the total cohort). Use of herbal alternatives was reported in two patients (20% of poorly adherent patients; 6.7% of the total cohort). Fear of medication and forgetfulness were each reported in one patient (10% of poorly adherent patients; 3.3% of the total cohort). Behavioral and cultural factors among poorly adherent patients are summarized in Table 3.

**Table 3:** Behavioral and cultural factors among poorly adherent patients Percentages are presented both among poorly adherent patients and in the total cohort. Poor adherence was defined as taking less than 80% of prescribed mesalazine doses

Factor	Number among poorly adherent patients (n = 10)	Percentage among poorly adherent patients	Percentage in total cohort
Stopping treatment when asymptomatic	6	60%	20%
Use of herbal alternatives	2	20%	6.7%
Fear of medication	1	10%	3.3%
Forgetfulness	1	10%	3.3%

Pill burden and dosing frequency

High pill burden, defined as four or more tablets per day, was observed in 16 patients (53.3%). Once-daily mesalazine was prescribed in six patients (20%), whereas 24 patients (80%) received multiple daily dosing regimens. Pill burden and dosing regimen are presented in Table 4.

**Table 4:** Pill burden and dosing regimen High pill burden was defined as taking four or more mesalazine tablets per day

Treatment-related factor	Number of patients	Percentage
High pill burden, ≥4 tablets/day	16	53.3%
Once-daily mesalazine regimen	6	20%
Multiple daily dosing regimen	24	80%

Financial and health system barriers

Financial constraints limiting regular mesalazine purchase were reported by eight patients (26.7%). Drug unavailability was reported by one patient (3.3%). These findings suggest that structural barriers may contribute to non-adherence in addition to patient-related behavioral factors. Financial and health system barriers are shown in Table 5.

**Table 5:** Financial and health system barriers Financial constraints were defined as difficulty purchasing mesalazine regularly because of cost. Drug unavailability was defined as patient-reported difficulty obtaining the prescribed formulation

Barrier	Number of patients	Percentage
Financial constraints limiting regular purchase	8	26.7%
Drug unavailability	1	3.3%

Response to targeted counseling

Among the 10 patients with poor adherence, seven patients (70%) improved their adherence after targeted counseling, while three patients (30%) had no documented improvement. This suggests that patient education may be a practical intervention in real-life clinical settings, particularly when nonadherence is related to misunderstanding of maintenance therapy or stopping medication after symptom improvement. Response to targeted counseling among poorly adherent patients is summarized in Table 6.

**Table 6:** Response to targeted counseling among poorly adherent patients Targeted counseling focused on explaining the chronic relapsing nature of UC, the role of mesalazine as maintenance therapy, the risk of relapse after treatment interruption, and the importance of regular dosing

Outcome	Number among poorly adherent patients (n = 10)	Percentage
Improved adherence after targeted counseling	7	70%
No documented improvement after targeted counseling	3	30%

## Discussion

This study describes mesalazine nonadherence in a Moroccan cohort of adults with mild-to-moderate UC. The main finding was that 10 of 30 patients (33.3%) had poor adherence, defined as taking less than 80% of prescribed doses. Non-adherence was not explained by a single factor. Instead, it reflected an interaction between patient behavior, cultural beliefs, pill burden, financial constraints, and medication access.

The observed poor adherence rate of 10 of 30 patients (33.3%) is consistent with the broader literature, where nonadherence to 5-ASA therapy in UC is commonly reported in routine practice [3,4,6]. A recent systematic review and meta-analysis found that nonadherence to oral mesalamine remains frequent and varies according to study design, setting, geographic area, and adherence assessment method [6]. The similarity between our findings and international data suggests that non-adherence is a universal challenge in UC management, although the underlying reasons may differ according to healthcare context.

Stopping treatment when asymptomatic was the most frequent behavioral factor in our cohort, reported in six of 10 poorly adherent patients (60%). This finding highlights a key challenge in chronic disease management: patients may interpret clinical remission as cure and may not understand the preventive role of maintenance therapy. Previous studies have shown that nonadherence to mesalazine in quiescent UC is associated with worse outcomes, including increased relapse risk [4,5]. Therefore, patient education should emphasize that symptom disappearance does not mean disappearance of relapse risk.

Cultural beliefs also contributed to nonadherence. Two of 10 poorly adherent patients (20%) used herbal alternatives, reflecting a practice that may be encountered in Morocco and other settings where traditional remedies are commonly used. The issue is not only the use of herbal products, but also their use as substitutes for maintenance therapy. Clinicians should ask about alternative remedies in a nonjudgmental way, because patients may not spontaneously disclose them. A culturally sensitive discussion may improve trust and help patients understand that complementary practices should not replace prescribed maintenance treatment.

High pill burden was another important finding. Sixteen of 30 patients (53.3%) received at least four tablets per day, and 24 of 30 patients (80%) were on multiple daily dosing regimens. Complex regimens may reduce adherence, especially in patients who are asymptomatic, professionally active, or financially constrained. Several studies have evaluated once-daily 5-ASA regimens and suggested that simplified dosing can maintain efficacy and may improve convenience or satisfaction [9-11]. Patient preference and adherence are also important determinants of long-term 5-ASA effectiveness, while oral 5-ASA remains a well-established maintenance therapy for UC [12,13]. In our setting, however, once-daily formulations were used in only six of 30 patients (20%), probably reflecting availability and affordability barriers.

Financial constraints were reported by eight of 30 patients (26.7%). This is particularly relevant in chronic diseases requiring long-term treatment. Even when a medication is clinically recommended, adherence may remain poor if the patient cannot purchase it regularly. In Morocco and similar healthcare environments, medication affordability should therefore be considered a central component of adherence assessment. A practical adherence strategy should include discussion of cost, prescription of accessible formulations when possible, and efforts to improve continuity of drug supply.

Drug unavailability was less frequent than financial limitation, affecting one of 30 patients (3.3%). However, even occasional interruption in medication access can contribute to treatment discontinuity. Availability problems may also encourage patients to change dose, skip tablets, or rely on alternative remedies. This reinforces the need for health system-level solutions in addition to physician-level counseling.

A clinically relevant finding was that targeted counseling was followed by improved adherence in most poorly adherent patients. This observation suggests that patient education could be helpful in real-life clinical settings; however, it should be interpreted cautiously because the study was descriptive, retrospective, and not designed to formally evaluate the effectiveness of an intervention. Educational and behavioral interventions tailored to individual patient barriers have previously been proposed as strategies to improve medication adherence in UC [7,8]. Counseling should be structured, repeated, and adapted to the patient’s specific barrier.

The study has several strengths. It provides real-life data from a Moroccan cohort, a population for which adherence data remain limited. It also explores several dimensions of adherence rather than focusing only on dosing behavior. By including behavioral, cultural, therapeutic, financial, and health system factors, the study reflects the complexity of adherence in daily practice.

However, several limitations should be acknowledged. First, the sample size was small, with only 30 patients, limiting generalizability. Second, the retrospective design may have led to missing or incompletely documented adherence determinants. Third, adherence was assessed using patient-reported information rather than objective methods such as pharmacy refill records, pill counts, electronic monitoring, or urinary 5-ASA metabolite testing. In addition, the study did not include comparative analyses between adherent and nonadherent patients, which limits the ability to identify factors independently associated with poor adherence. The evaluation of adherence improvement after targeted counseling was also based on documented follow-up information and clinician assessment rather than a standardized adherence questionnaire. Fourth, the study did not evaluate clinical outcomes such as relapse, corticosteroid use, hospitalization, mucosal healing, or quality of life. Finally, the improvement after counseling was descriptive and should be interpreted cautiously because there was no control group and no standardized long-term adherence assessment.

Despite these limitations, the study highlights practical adherence barriers that are highly relevant to routine UC care in Morocco. Future prospective studies using validated adherence scales and objective adherence measures are needed. Larger multicenter studies could better define the relative contribution of cost, formulation availability, patient education, and cultural beliefs to mesalazine adherence in North African populations.

## Conclusions

Mesalazine nonadherence was frequent in this Moroccan cohort of patients with mild-to-moderate UC. The main contributors were treatment interruption during asymptomatic periods, use of herbal alternatives, fear of medication, forgetfulness, high pill burden, financial constraints, and occasional drug unavailability. These findings show that nonadherence is not only a patient behavior issue, but also a therapeutic, socioeconomic, cultural, and health system challenge.

Improving adherence requires a practical, patient-centered approach. Strategies may include repeated education on the chronic nature of UC, explanation of the preventive role of maintenance therapy, simplification of dosing regimens when feasible, consideration of drug affordability, and improved access to mesalazine formulations. In this cohort, targeted counseling was followed by improved adherence in most poorly adherent patients, supporting the potential value of structured education in routine clinical practice. However, this finding should be interpreted cautiously because the study was not designed to formally assess the effectiveness of counseling.
